# Breast Cancer Care in Jordan

**DOI:** 10.1200/JGO.19.00279

**Published:** 2020-02-21

**Authors:** Hikmat Abdel-Razeq, Asem Mansour, Dima Jaddan

**Affiliations:** ^1^ Department of Internal Medicine, Section of Hematology and Medical Oncology, King Hussein Cancer Center, Amman, Jordan; ^2^ School of Medicine, University of Jordan, Amman, Jordan; ^3^ Department of Radiology, King Hussein Cancer Center, Amman, Jordan

## Abstract

Breast cancer is the most common malignancy in Jordan and the third leading cause of cancer death after lung and colorectal cancers. Although the incidence of breast cancer in Jordan is lower than that in industrialized nations, the number of new cases has been significantly increasing, and women present with breast cancer at a younger age and with more advanced disease than women in Western countries. Jordan is a medium-income country with limited resources and a young population structure. Therefore, breast cancer poses a particularly challenging burden on the country’s health care system. Despite ongoing endeavors to improve breast cancer care at both public and private levels, more work is needed to achieve downstaging of the disease and improve access, awareness, and participation in early detection. Multimodality treatment facilities and supportive care are available; however, the quality of care varies widely according to where the patient is treated, and most treatment facilities remain located centrally, thus, creating access difficulties. The King Hussein Cancer Center, the only comprehensive cancer center in Jordan, has changed the practice of oncology in the country via implementation of a multidisciplinary approach to treatment, monitoring of treatment outcomes, and investments in ongoing cancer research. However, there remains no national system for ensuring provision of high-quality cancer care nationwide. Here, we review the epidemiology of breast cancer and the current status of breast cancer care in Jordan, we compare our treatment outcomes with international ones, and we highlight challenges and improvement opportunities.

## INTRODUCTION

Globally and in the Eastern Mediterranean Region (EMR), breast cancer is the most common malignancy and the leading cause of cancer death among women.^1,2^ In 2018, there were around 2.1 million new breast cancer occurrences and 626,679 deaths worldwide.^1^ In Jordan, cancer is the second leading cause of death after cardiovascular disease, and breast cancer is the third most common cancer death after lung and colorectal cancers.^3^ Data on breast cancer pathology, clinical presentation, and treatment outcomes in the region are very limited and mostly represent retrospective analyses. Recruitment to clinical trials is very limited, and patients are notably underpresentable.^4^

Jordan is an Arab country located in the EMR with a land area of approximately 89,000 km^2^. It is home to 10 million inhabitants and has an annual population growth rate of 2.4%.^5^ The country has a young population structure; in 2017, 34.3% of the population was younger than age 15 years, 62% were between age 15 and 65 years, and 3.7% were older than age 65 years.^5^ Life expectancy at birth has notably improved over the last 50 years, from approximately 58.0 years in 1967 to 74.5 years in 2017.^6,7^

According to the World Bank, Jordan is an upper middle-income country.^7^ In 2017, the gross domestic product was estimated at $40 billion, with an annual growth rate of 2.0%.^7^ In 2014, health care constituted approximately 7.5% of gross domestic product expenditure.^8,9^ Jordan has one of the most advanced health care systems in the Arab region, which attracts patients for treatment from several neighboring countries. The country’s health care is a two-tiered system provided through the public and private sectors. The public sector is composed of the Ministry of Health (MOH), the military’s Royal Medical Services, and 2 university hospitals. The private sector runs 62 hospitals that account for approximately 33% of all hospital beds.^10^

Cancer treatment modalities are available in many private and public hospitals as well as at the King Hussein Cancer Center (KHCC), a nongovernmental, nonprofit cancer tertiary center founded in 1997 that currently treats approximately 60% of cancer occurrences in Jordan and provides cancer treatment to patients from several countries in the surrounding region.^11^ Cancer treatment is not covered by private insurance, so the government bears the expenses of treating Jordanian patients with cancer at public hospitals and at the KHCC. Because structured primary care programs and services are lacking in the whole country, following patients with cancer who have treatment-related complications or even comorbidities while undergoing anticancer therapy and beyond puts a lot of pressure on cancer centers to provide such service.

**CONTEXT**

**Key Objective**
Despite a significant decline in the incidence of breast cancer in the West, breast cancer in Jordan and the region is on the rise. There is a paucity of regional real-world, high-quality data on breast cancer. Addressing the current status of breast cancer in Jordan should help clinicians, researchers, and decision makers in the region better understand unmet needs.
**Knowledge Generated**
Investing in national cancer control plans to increase awareness and early detection of breast cancer, as well as improving infrastructure and equipment, high-quality medical education and training, national clinical practice guidelines, and multidisciplinary care paired with well-defined quality and accreditation programs in addition to clinical research, should be part of national strategies to deal with the increasing burden of breast cancer.
**Relevance**
Findings highlighted here reflect the current status of breast cancer care in most neighboring countries and should help understand the disease landscape in the region for additional improvement of patient care.

The King Hussein Cancer Foundation has changed the insurance landscape for patients with cancer by introducing a nonprofit cancer insurance program that covers the cost of cancer care at KHCC for program participants who pay affordable premiums.

Here, we describe the epidemiology of breast cancer and the current status and quality of breast cancer care in Jordan. In a country with limited resources, it is imperative to understand the current status of cancer care in Jordan to improve decision making and resource allocation.

## MATERIALS AND METHODS

We used data from the latest annual report published by the Jordan Cancer Registry (JCR) in 2015.^12^ The JCR is a population-based cancer registry established in 1996 under the jurisdiction of the Ministry of Health (MOH). Cancer occurrences can be registered only with a tissue diagnosis, and reporting is compulsory. Malignant and in-situ cancer occurrences in Jordan are reportable to the JCR; therefore, all cancers diagnosed or treated in Jordan since 1996 are registered in the JCR database.^12^ However, the registry does not collect data on cancer survival or treatment outcomes. For analysis of treatment outcomes, we used data from the cancer registry at the KHCC, which is a hospital-based registry established in 2006 that currently includes > 44,000 cancer occurrences diagnosed, treated, and followed at the institution since 2006; this collection represents almost two thirds of the cancer burden in the country (unpublished data). All consecutive patients with pathologically proven diagnoses of breast cancer confirmed, treated, and followed in our institution were included in the survival analysis. Vital status at the time of data analysis was obtained through direct connection with the Department of Civil Status, a governmental agency that issues all death certificates.

Follow‐up duration was calculated from the date of diagnosis until the date of death or last clinical follow-up. The median follow‐up time was 57 months (range, 15-158 months). Survival curves were presented using the Kaplan-Meier method, and the significance of differences in median survival duration between groups was assessed using the log-rank test. Overall survival (OS) was estimated from the date of diagnosis to the date of death as a result of any cause.

To compare our breast cancer rates with regional and international ones, we used data from the Global Burden of Disease (GBD) for cancer incidence and mortality in the year 2015.^2,13^ For studies of breast cancer in Jordan, Pubmed/Medline was searched using variations of the following key words: breast, cancer, and Jordan.

## EPIDEMIOLOGY OF BREAST CANCER

**FIG 1:**
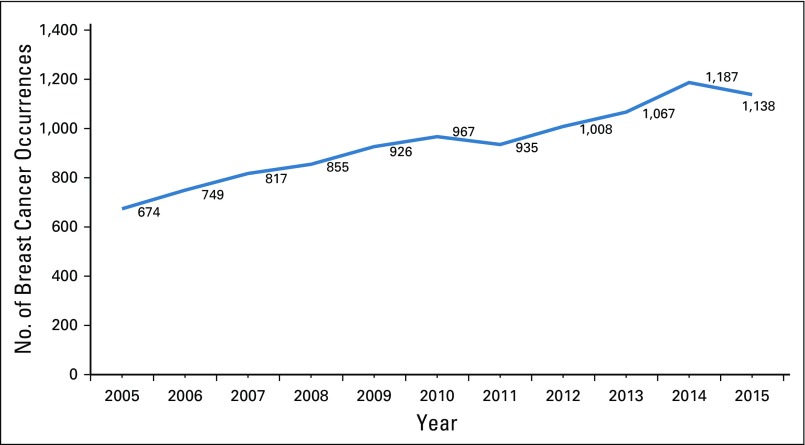
Number of breast cancer occurrences (2005-2015).

**FIG 2:**
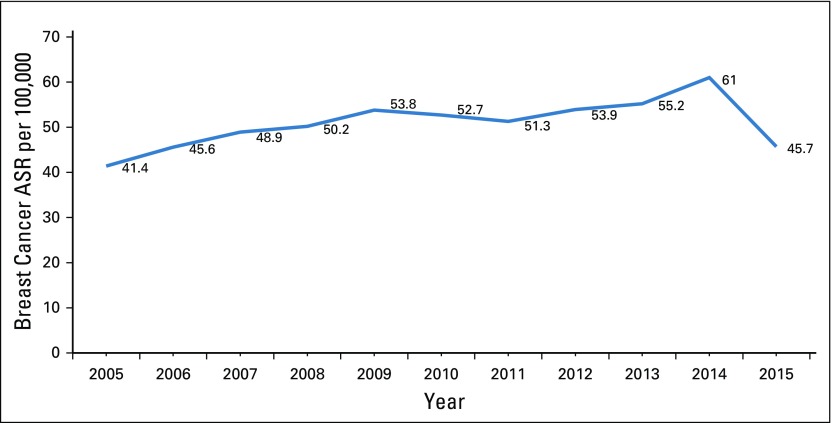
Breast cancer age-standardized rate (ASR) per 100,000 (2005-2015).

**FIG 3:**
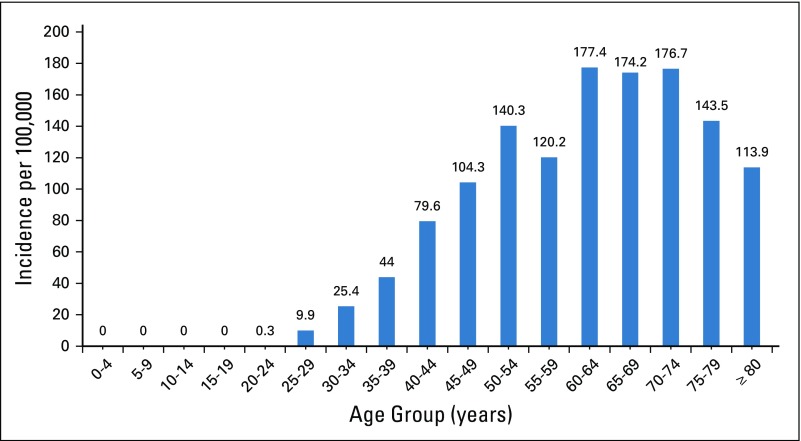
Age-specific incidence rates per 100,000 (2015).

According to latest statistics from the JCR, breast cancer is the most common cancer in Jordan, accounting for 20.6% of cancers in Jordanians of both sexes and 39.4% of cancers among Jordanian women.^12^ The number of breast cancer occurrences in women has increased by 69% during the past decade, from 674 in 2005 to 1,138 in 2015 (Fig 1). The crude incidence of breast cancer among Jordanian women in 2015 was 34.1 per 100,000, whereas the age-standardized incidence rate (ASR) was 45.7 per 100,000, which is lower than the ASR of 61.0 per 100,000 reported in the previous year (Fig 2). Approximately 15.6% new patients with breast cancer were younger than age 40 years; 29.1% were between 40 and 49 years; 25.6%, between 50 and 59 years; and 29.7%, older than age 60 years. Age-specific rates for breast cancer in women for 2015 (Fig 3) show the highest incidence of 177.4 per 100,000 in the age group 60 to 64 years, followed by 176.7 per 100,000 in women between age 70 age 74 years. The median age at diagnosis among women was 51 years, which is approximately 10 years younger than the median age at diagnosis of breast cancer for women in Western countries.^14,15^

**FIG 4:**
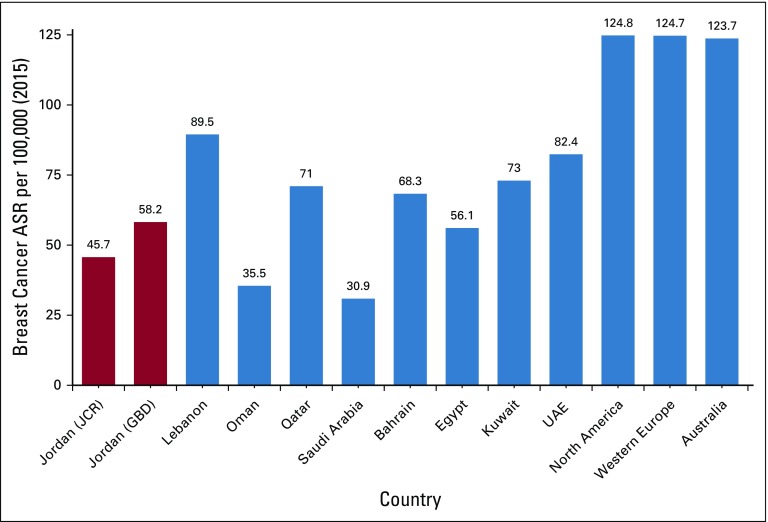
Breast cancer age-standardized rate (ASR) per 100,000 in 2015; GBD, Global Burden of Disease; JCR, Jordan Cancer Registry; UAE, United Arab Emirates.

Figure 4 compares female breast cancer ASRs in 2015 among Jordan, other EMR Arab countries, North America, Western Europe, and Australia (data from GBD 2015). Of note, ASR rates for Jordan estimated by JCR differ from those based on the GBD study (45.7 *v* 58.2 per 100,000, respectively). Nevertheless, and given the limitations of GBD data, rates observed for Jordan are lower than those in other countries except for Saudi Arabia, Egypt, and Oman.

## BREAST CANCER SCREENING AND EARLY DETECTION

In an effort to reduce mortality and morbidity from breast cancer, a national breast cancer–specific screening and early detection program, the Jordan Breast Cancer Program (JBCP), was established under the directive of the MOH in 2006. Before the setup of the JBCP, breast cancer care has been focused largely on treatment, as > 70% of patients were being diagnosed with advanced disease. Thus, the JBCP was established to provide universal access to screening mammography for all women age ≥ 40 years. However, because of limited resources, the strategy has shifted toward early detection and downstaging of breast cancer. One of the main gaps in providing screening services was the shortage of specialized radiologists and lack of female trained technicians. Therefore, the JBCP facilitated capacity building by investing in human power and conducting national training workshops for radiologists, radiographers, nurses, and oncologists.^16^ The JBCP has set national diagnostic and screening guidelines that currently are followed by many health care practitioners.^17^ Although many international guidelines recommend screening mammography after the age of 45 or 50,^18,19^ the JBCP recommends the commencement of screening mammography at the age of 40.^16,17^ This is because of the younger age of onset in Jordan: 50% of breast cancer among Jordanian women occurs before age 51.^12^

Despite progress, there have been several barriers to achieving organized population-based screening in Jordan, including limited resources and various challenges facing the screening program. In an effort to improve access to screening for women living in underprivileged areas, the JBCP currently provides two mobile mammography units that are centrally connected to the KHCC.^16^ However, ensuring periodic screening of women who have been screened still remains a challenge.

Research on Jordanian women’s knowledge of breast cancer and their attitudes and barriers toward screening is scarce. The few available studies about knowledge, attitudes, and practices have revealed limited knowledge about breast cancer and breast cancer screening as well as low participation rates in early detection practices (clinical breast examination rates between 17% and 28% and screening mammography rates of 7%-9%).^20,21^ In a cross-sectional study of 327 Jordanian women diagnosed with breast cancer between 2012 and 2014, around 32.2% of patients sought medical care > 3 months after the discovery of a breast cancer symptom, and 65.5% of the women attributed the delay in seeking medical advice to their ignorance of the nature of the problem. The median time between observing a symptom and seeking medical advice was 30 days, which is higher than the median time in high-income countries (7-16 days).^22^ The JBCP addresses these deficiencies by conducting yearly awareness campaigns and educational programs to improve public awareness about breast cancer.^16^ Although studies reported improvements in women’s knowledge after these educational sessions,^23,24^ their effect on women’s participation in screening is yet to be determined.

## DIAGNOSIS AND STAGING

There are > 60 mammography units nationwide,^25^ and several private hospitals offer 3- dimensional digital breast tomosynthesis. With the wider availability of mammography screening, more women are undergoing breast biopsies. Chest x-ray, magnetic resonance imaging, computed tomography scanning, bone scans, and positron emission tomography scans are widely available for staging of breast cancer as well as for monitoring response to therapy and surveillance of survivors. However, few institutions adhere to national diagnostic guidelines^17^ and implement multidisciplinary meetings in diagnosis and staging.

## STAGE AT DIAGNOSIS AND PATHOLOGIC EVALUATION

As reported by the national cancer registry, fewer than one third of women in Jordan present with localized disease at the time of diagnosis, and 13.4% present with metastatic disease.^12^ In comparison, 6% in the United States and the United Kingdom present with distant metastasis.^26,27^ The majority (84.7%) of patients treated at KHCC between 2015 and 2017 were estrogen receptor and/or progesterone receptor positive; 8.3% had triple negative (TN) disease, and 17.7% were HER2/neu positive (unpublished data). Regionally, there is notable variation in the percentage of metastatic disease at diagnosis; such a rate is as low as 6.0% in Lebanon.^4^

## GENETIC TESTING

According to limited published data, almost 10%-15% of breast cancer occurrences are inherited; *BRCA1* and *BRCA2* gene mutations are identified as the most common genetic alterations found in patients with breast cancer.^28^ Although testing for mutations in high-penetrance genes, such as *BRCA1* and *BRCA2*, has become standard practice for patients with breast cancer in many places in Western countries,^29^ genetic screening is not routinely performed in most Jordanian hospitals. Therefore, little is known about the genetic composition of *BRCA1*/*2* mutations among Jordanian patients. Our group recently published our first experience with *BRCA1*/*2* research among 100 high-risk patients with breast cancer in Jordan. Results showed that 20% of patients had deleterious mutations and that 7% had suspected deleterious mutations in *BRCA1* or *BRCA2* genes. Highest mutation rates were observed among patients with TN status (56.3%), especially among those with a positive family history of breast and/or ovarian cancer (69.2%) and patients with bilateral or second primary breast cancer (66.7%).^30^ In a subsequent larger study of 500 high-risk patients with breast cancer treated at our institution, approximately 13% of the whole group had pathogenic/likely pathogenic *BRCA1*/*2* variants, and 8.8% had variants of uncertain significance. The prevalence of pathogenic/likely pathogenic *BRCA1*/*BRCA2* gene mutations among patients with TN disease was 33.9%, and it was 60% among patients with both TN disease and a family history of breast cancer.^31^

To address the implications of these results, a genetic counseling clinic was established at the KHCC in 2015. Initially, genetic testing was limited to high-risk groups and direct family members, but it has gradually expanded, and services currently are available to a wider population, as per the National Comprehensive Cancer Network guidelines.^32^ Such high-risk patients include all those diagnosed at age ≤ 40 years; all patients younger than age 50 who have one or more close relative(s) with breast cancer; those younger than age 60 with TN breast cancer (negative for estrogen receptors, progesterone receptors, and HER2 receptors); and those diagnosed at any age with ≥ 2 close relatives with breast cancer, with ≥ 1close relative(s) with invasive ovarian cancer, or with a close male relative with breast cancer.

Given the financial limitations, testing beyond *BRCA1*/*2* is not routinely done in clinical practice. However, a study is ongoing to test for wider genetic mutations in patients who tested negative for *BRCA1*/*2*.

## TREATMENT AND SUPPORTIVE CARE

### Surgery

More than half of patients with breast cancer in Jordan have their surgery done by specialized surgical oncologists; > 10 of them are practicing at the KHCC, in the university hospitals, and in the private sector. However, in many cases, breast cancer initially comes to the attention of a general surgeon in a secondary hospital, where breast surgery is performed. This sometimes leads to suboptimal outcomes, such as positive margins, partial removal of the breast mass, or inadequate management of the axilla. Sentinel lymph node biopsies are offered in only a few centers. Thus, full axillary lymph node dissection is still widely practiced and results in lymphedema in 21.1% of women treated for breast cancer in Jordan.^33^ Skin-sparing and nipple-sparing procedures are limited to one center only. Likewise, reconstructive surgery is not widely available and is not covered by cancer insurance. Waiting time for surgery is very variable but usually is not an issue nationwide.

### Anticancer Drugs

A large number of chemotherapeutic agents, including modern ones, and targeted therapy are widely available across all medical centers despite their high costs. Trastuzumab, a HER2-specific monoclonal antibody, was introduced shortly after its approval by the US Food and Drug Administration and has been used across its indications in the neoadjuvant, adjuvant, and metastatic settings. Another HER2-specific monoclonal antibody, pertuzumab, was in use for many years in the neoadjuvant setting and recently has been approved for use in the metastatic setting. Because most of our patients present with node-positive disease and larger tumor size, more than half of our patients with nonmetastatic disease receive neoadjuvant chemotherapy. Most endocrine therapy drugs, including fulvestrant, are available and covered by cancer insurance as well. Endocrine resistance modulators, such the mTOR kinase inhibitor everolimus and the CDK4/6 inhibitors palbociclib and ribociclib, are available in big public and private sectors, too. Supportive care during cancer treatment (ie, colony-stimulating factors, antiemetics, and intensive care), when needed, is widely available.

### Radiotherapy

As per international guidelines, and similar to what patients with breast cancer can get in more developed countries, radiation therapy is offered as an adjuvant treatment after surgery in women with high risk of locoregional recurrence, including those with a tumor size ≥ 5.0 cm, positive lymph nodes, positive resection margin(s), and those undergoing breast-conserving surgery. There are 6 linear accelerators (Versa HD [Elekta, Stockholm, Sweden] with advanced properties) at the KHCC, 2 linear accelerators at the MOH, 3 at military medical services, 2 in a private hospital, and two more at the King Abdullah Hospital in the northern part of the country; the total, then, is 15 accelerators, or one accelerator per 600,000 people. Because most of the radiation therapy services are centrally located, support for transportation and accommodation is offered to rural patients who require treatment. Computed tomography simulator-based forward-planned intensity-modulated radiotherapy planning is used regularly at the KHCC with either conventional or hypofractionated whole-breast irradiation. Neither partial breast irradiation techniques, including breast brachytherapy, nor stereotactic body radiotherapy is available.

### Palliative and End-of-Life Care

In the 1990s, end-of-life care was provided by small hospice care centers, such as the AlMalath foundation in Amman. After launching the Jordan Palliative Care initiative in 2001, a palliative unit was established at the KHCC.^34^ The department consists of a specialized palliative team that coordinates with the oncology team. Early referral to the palliative program is strongly encouraged and monitored as a quality key performance indicator. The program provides inpatient and outpatient services as well as the largest home care program in the country.^35^

In 2017, the Jordan Medical Council approved palliative care as a medical specialty, thus opening the door for new training programs and availability of palliative care specialists. In addition, legislative and regulatory changes have been implemented to specifically improve access to opioids, such as increasing the number of days for which opioids can be prescribed and the production of low-cost, generic, immediate- release morphine tablets.^36^

### Survivorship Program

With the recent improvements in breast cancer treatment and the wider implementation of early detection and screening programs, more women are surviving breast cancer. After the completion of their treatment, many survivors need follow-up care and support to enable them to manage the adverse effects of cancer and its treatment. Several countries have issued recommendations and guidelines for survivorship care, which usually is provided by primary care providers.^37^

Primary care medicine does not exist in Jordan, so a breast cancer survivorship program was established at the KHCC to provide follow-up care for survivors within an institutional setting. The survivorship clinic is designed to ease the transition from treatment to normal life while offering evidence-based care. In coordination with the primary oncology team, the survivorship team develops a personalized care plan for each patient. This plan includes reviewing the patient’s medical history and performing a physical examination; screening for breast cancer recurrence or new primary cancer diagnosis; and identifying and helping survivors manage adverse effects of cancer and its treatment, such as fatigue, osteopenia, menopausal symptoms, and sexual problems. To prevent treatment-related bone loss, women receive calcium and vitamin D supplementation and receive advice and referrals for lifestyle interventions, such as exercise and smoking cessation. Women at high risk of fracture are given bisphosphonates or denosumab.

### Accreditation and Quality of Care

Voluntary hospital accreditation in Jordan is granted by the Health Care Accreditation Council, which is a national agency that establishes health care quality standards for hospitals, breast imaging units, and other health care facilities.^38^ Almost 30 hospitals are accredited by the Health Care Accreditation Council in Jordan, including 7 private hospitals, 2 university hospitals, and the KHCC.^39^ Several hospitals, including the KHCC, also are internationally accredited by the Joint Commission International as hospital programs.^40^ In addition, the KHCC is the only hospital in Jordan that has been awarded the Joint Commission International Clinical Care Program Certificate for its oncology program.^40^

In Europe and the United States, specialized accreditation programs have been established to ensure the delivery of quality care at breast units. The European Society of Breast Cancer Specialists identified 17 quality indicators, 10 of which are used for certification purposes.^41^ Similarly, in the United States, the National Accreditation Program for Breast Centers developed key standards required for National Accreditation Program for Breast Centers accreditation.^42^ Chief among the process quality indicators for breast cancer care required by most certification programs in Europe and the United States is the multidisciplinary team meeting.^43,44^ At the KHCC, a multidisciplinary team was established that meets twice weekly. Team members are mandated to follow established clinical practice guidelines, and a peer-review committee that reports to a quality office and the chief medical officer was established to monitor adherence with these guidelines.

### Treatment Outcomes and Survival Estimates

Breast cancer treatment outcomes are not available at the national level. However, such data are available at an institutional level at the KHCC, where effort is made to constantly monitor and evaluate outcomes and benchmark them against international figures.

**FIG 5:**
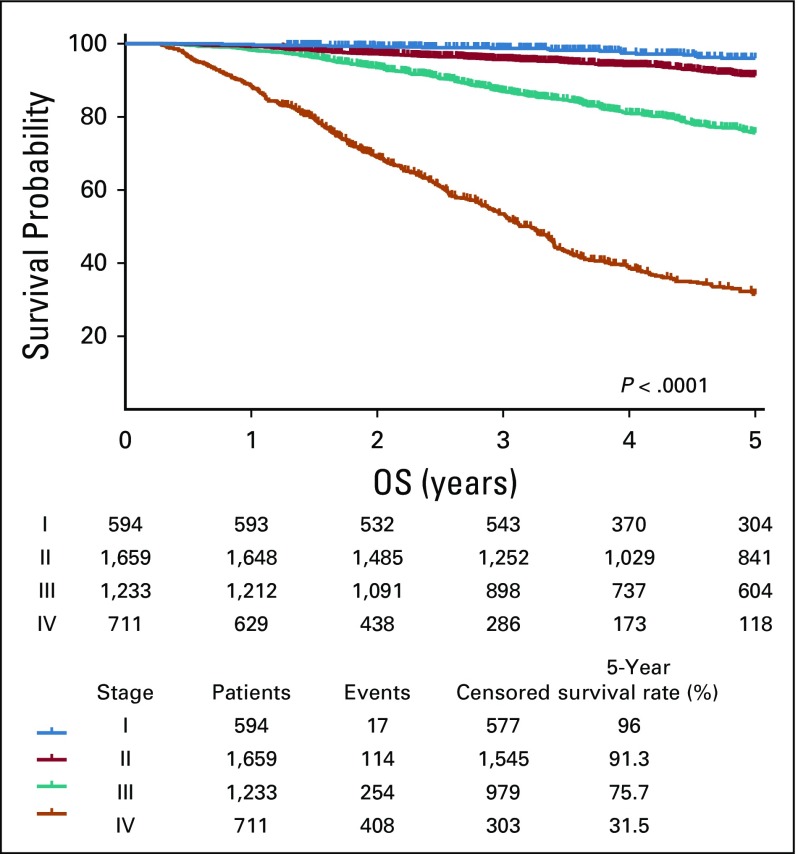
Overall survival probability.

Survival analysis of 4,561 patients with breast cancer treated and followed at our institution between July 2006 and December 2017 revealed 5-year survival rates of 96% for patients with stage I disease, 91.3% for stage II, 75.7% for stage III, and 31.5% for stage IV. There was a significant difference in survival rates according to stage at diagnosis (*P* < .0001; Fig 5).

At our institution, we also recently published outcome results assessing the use of neoadjuvant chemotherapy in patients with breast cancer. In a retrospective study of 346 patients treated between 2003 and 2014 using the National Surgical Adjuvant Breast and Bowel Project Protocol B-27 (NSABP-B27) chemotherapy regimen (in which four cycles of doxorubicin and cyclophosphamide followed by four cycles of docetaxel are given preoperatively), pathologic complete response, defined as no evidence of invasive carcinoma in the breast at the time of surgery, was achieved in 25% of patients.^45^ Such rate is comparable to that reported in the NSABP-B27 trial (26%), although our patients had larger average tumor size (6.0 cm *v* 4.5 cm) and more axillary lymph node involvement (78.3% *v* 30.5%). However, the proportion of patients receiving breast conservation surgery in our study was much lower than that reported in the original trial (24.6% *v* 63.7%, respectively).^46^ In addition, our 5-year disease-free survival (DFS) of 63.5% was less than that achieved in the NSABP-B27 trial (71.1%).^47^ These differences could be attributed to worse tumor characteristics in our patients.

In another study, data from 121 patients with HER2-positive breast cancer treated at the KHCC between 2008 and 2015 with the NSABP-B27 neoadjuvant chemotherapy regimen and trastuzumab were reviewed. After neoadjuvant therapy, pathologic complete response was achieved in 49.6% of the patients who underwent surgery.^48^ The 3-year DFS and OS rates were 84.2% and 87.2%, respectively, which were comparable to those reported in large clinical trials, such as the German TECHNO trial (3-year DFS and OS rates of 77.9% and 89.4%, respectively) and the NOAH trial (3-year OS of 87%).^49,50^

### Breast Cancer Research

Breast cancer research in Jordan has been relatively limited. A search of PubMed yielded a total of 105 publications between 1985 and 2019. Only 3 papers were published between 1985 and 1999, whereas 82 papers were published between 2010 and 2019. Data are still relatively scarce, particularly with regard to survival and treatment outcomes; breast cancer genetics; trends in risk factors; quality of care; and other aspects of breast cancer care, such as survivorship and palliative care. At the KHCC, these deficiencies are being addressed via efforts to enhance research and establish collaborative research agreements with international groups. For example, the KHCC has a partnership agreement with the University of Texas MD Anderson Cancer Center, which has facilitated several research projects in breast cancer and palliative care through the center’s Sister Institution Network Fund.^51^ Also, the KHCC has recently joined the European Organization for Research and Treatment of Cancer, which has a network of > 5,300 scientists across 37 countries.^52^ In addition, the country had participated in many international randomized clinical trials and studies.

In conclusion, the incidence of breast cancer in Jordan is lower than that in industrialized nations. Nevertheless, it is the commonest malignancy, and the number of new occurrences has been increasing greatly. In addition, women present with breast cancer at a younger age and with more advanced disease than women in Western countries. Despite ongoing endeavors to improve breast cancer care on both public and private levels, more work is needed to achieve downstaging of the disease and improve awareness and participation in early detection and screening. Multimodality treatment facilities and supportive care are available; however, they remain largely located centrally, which poses access difficulties for women living in the south and rural areas. Quality of care can be improved more by setting national quality standards for breast cancer care with monitoring of key process and outcome indicators. Additional efforts are needed to develop strategies that would facilitate the adoption of new diagnostic and treatment technologies and implementation of risk-stratified screening and tailored therapy.
